# A new technique to reposition an incarcerated gravid uterus based on a biomechanical concept

**DOI:** 10.1007/s00404-026-08404-4

**Published:** 2026-08-27

**Authors:** Bernd Morgenstern, Janice K. Jeschke

**Affiliations:** 1 Department of Gynecology and Gynecological Oncology, University Hospital Cologne and Medical Faculty, Cologne, Germany; 2 Department of Obstetrics and Gynecology, Faculty of Medicine, University Hospital Münster, University of Münster, Münster, Germany

**Keywords:** Incarcerated gravid uterus, Pregnancy complication, Repositioning, Minimally invasive

## Abstract

**Introduction:**

An incarcerated gravid uterus (IGU) is a rare but serious obstetrical complication. Currently, there is no consensus on the optimal treatment when conservative reduction techniques have failed. Often, maneuvers with increased force are applied or a surgical intervention is the final reserved measure. Both are associated with an increased risk for the mother and child. In this technical report, we present a novel, minimally invasive technique to reduce an incarcerated uterus based on a biomechanical concept.

**Method:**

To reduce an IGU, we apply saline fluid intraabdominally under sonographic guidance, while the patient is under peridural anesthesia. This not only increases the space in the abdomen necessary for repositioning, but also negates the negative pressure in the pouch of Douglas created when removing the uterine fundus from the true pelvis.

**Results:**

The author's experience with this method is limited to two cases, both resulting in successful uterine repositioning after failure of the usual conservative reduction techniques.

**Conclusion:**

This procedure provides a viable alternative for reducing an IGU, thereby minimizing the need for more invasive measures that carry considerably higher maternal and fetal risks.

## What does this study adds to the clinical work

**Table:** 

A novel, minimally-invasive procedure that can reposition an incarcerated gravid uterus with reduced risk for the mother and her pregnancy.	

## Introduction

Outside of pregnancy, the uterus usually lies in an anteflexed and anteverted position. In 11.2% of women, the uterus lies retroflexed or retroverted [1]. With increasing size of the uterus during pregnancy, a change in position of a retroflexed uterus almost always occurs spontaneously [2]. However, when a retroverted uterus does not reposition, the cervix can become anterosuperiorly displaced. The uterine fundus is then lodged within the true pelvis between the sacral promontory and the pubic symphysis, resulting in a condition called an incarcerated gravid uterus (IGU) [3, 4].

An IGU is an obstetrical complication that has been estimated to occur in 1 out of 3000 pregnancies [3]. There are several risk factors predisposing an IGU, such as intraabdominal adhesions, endometriosis, prior pelvic inflammatory disease, congenital anatomical abnormalities, pelvic tumors, uterine fibroids, and leiomyoma [5–7]. If not treated, an IGU can lead to severe obstetrical complications, such as intrauterine growth retardation, anterior uterine wall thinning or sacculation, uterus rupture, premature rupture of membranes, preterm labor, or even the loss of the pregnancy [7–9]. Potential maternal consequences can include severe outcomes, such as bladder rupture, kidney failure, or even death [3, 8, 9].

To date, there is no consensus on the optimal management of an IGU [7, 9]. As a rule, measures are taken which, if unsuccessful, lead to an escalation of force applied to the uterus or a surgical intervention [6]. The current techniques include applying downward traction on the uterus or transvaginal pressure upwards on the fundus, or both simultaneously, as well as surgically repositioning the uterus or terminating the pregnancy [10, 11]. These strategies increase the risk of intervention-related complications. In order to resolve an IGU, we apply a biomechanical concept to dislodge an entrapped, gravid uterus, if repositioning of the uterus using other conservative techniques was not successful.

### Methods

**Fig. 1:**
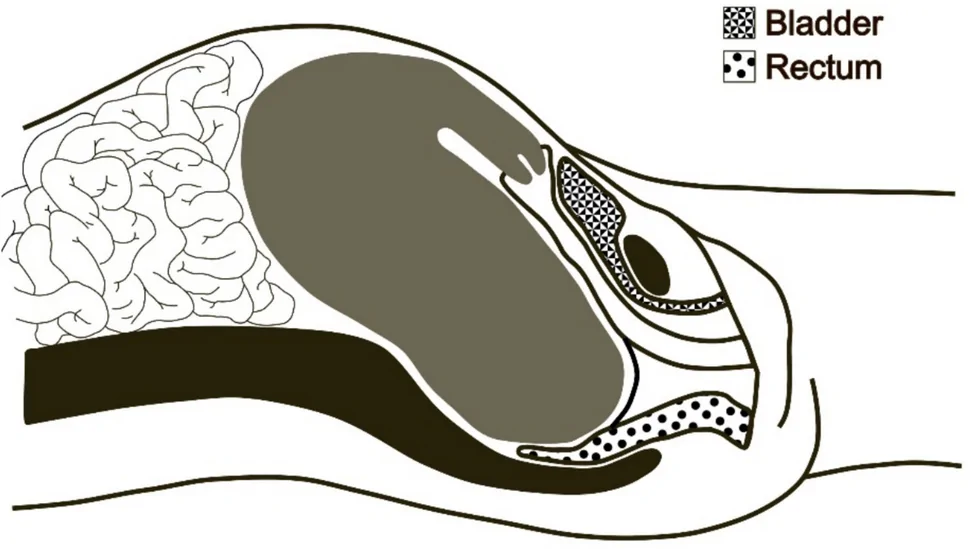
The initial IGU. Due to its fixed retroflexed position, the fundus of the uterus extends into the Douglas space. Consequently, the cervix is displaced cranially. Since no other intraabdominal organs, fluids or gases can fill the gap between the uterine fundus and the Douglas peritoneum, negative pressure develops when attempting to remove the fundus from the true pelvis

**Fig. 2:**
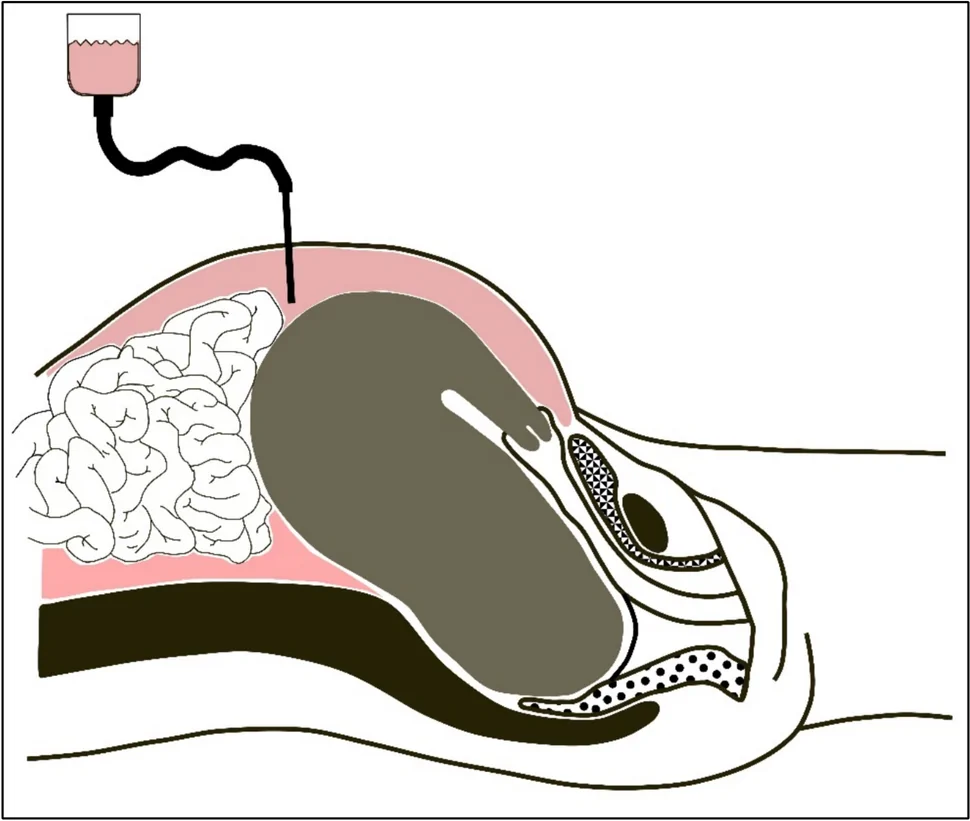
Intraabdominal fluid application. This figure depicts the introduction of warm saline solution (colored pink) into the abdomen via a Veres cannula. The infusion increases the distance between the spine and the ventral abdominal wall, which facilitates the subsequent rotation of the uterus. Sterile catheterization ensures safe bladder emptying

**Fig. 3:**
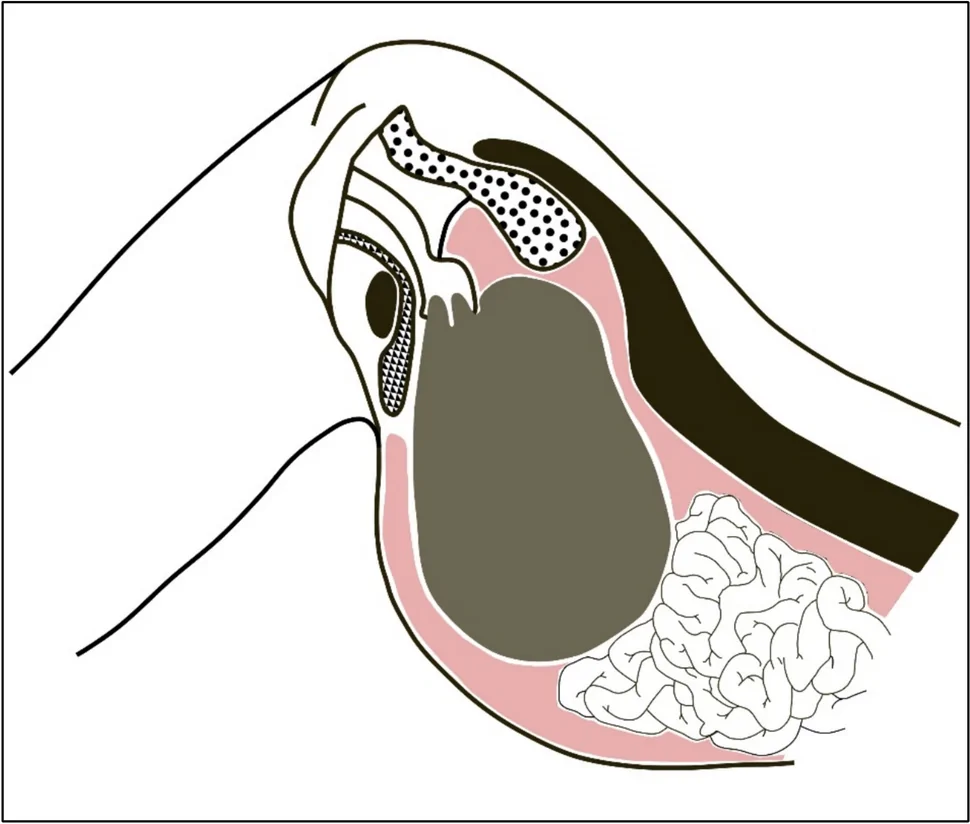
Repositioning the IGU. Following the removal of the Veres cannula, the patient is placed in a deep knee–elbow position with the pelvis elevated above the abdominal cavity. Transrectal digital pressure on the fundus induces the rotation of the uterus. During this procedure, four key factors work synergistically: 1. the additional intraabdominal fluid increases the distance between the abdominal wall and the spine; 2. the fluid fills the space between the uterine fundus and the Douglas peritoneum, thus preventing the formation of negative pressure when the fundus is displaced from the true pelvis; 3. manual pressure on the uterine fundus gradually displaces the fundus from the true pelvis; 4. gravity acts on the uterus in such a way that its weight facilitates rotation

**Fig. 4:**
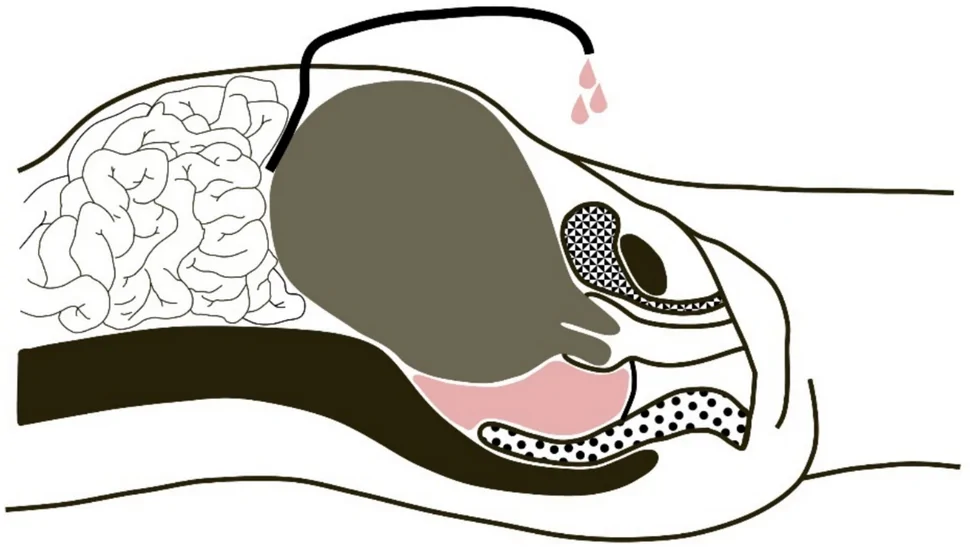
End of the procedure. After confirming the successful rotation of the uterus into its anatomical position via clinical palpation and transabdominal sonography, the patient is moved from the knee–elbow position back into the supine position. The artificially induced ascites is drained from the abdomen using a sonography-guided gravity-assisted silicone drain. Any remaining amounts of undrained fluid are reabsorbed from the peritoneal cavity. After completion of the procedure, the patient undergoes a period of continuous fetal monitoring

To facilitate the procedure and to reduce the tension of the maternal abdominal wall, a peridural anesthesia is applied without compromising the patient’s motor function. During the intervention, fetal well-being is continuously monitored using ultrasound. The technique, which is shown schematically in Figs. 1, 2, 3, and 4, begins with the application of local anesthesia, as well as a small skin incision in the upper left abdomen. Under sonographic guidance, a Veres cannula is used to puncture the peritoneal cavity. Warmed saline solution is then filled into the peritoneal cavity until the patient reports significant, yet tolerable abdominal pressure. Next, the patient’s urinary bladder is emptied through a sterile catheterization. As the patient is in a deep knee–elbow position, light digital pressure is applied to the uterine fundus transrectally to facilitate the rotation of the uterus to its anatomical position. If the digital pressure is not sufficient, the rectum can be filled. The repositioning of the uterus to the desired physiological position is confirmed and documented via ultrasound. A silicone catheter (suprapubic bladder catheter) is then inserted, under sonographic guidance and through a split cannula, at the site of the previous fluid filling. After the fluid has drained through the catheter, it is removed along with the epidural catheter. Finally, the patient’s bladder is drained via a urinary catheter until the effects of the regional anesthesia have worn off.

## Results

We had two patients admitted to our tertiary perinatal center with the diagnosis of an IGU (2017; 2023), where several conservative treatment options had not successfully repositioned the gravid uterus. Both patients were at a gestational age of 25 weeks. Due to the persisting symptoms, an escalation of the therapy was necessary. Before applying additional force or letting the patient undergo an extensive surgical procedure, we applied our new technique based on biomechanical principles. Volumes of 3.6 and 4.1 L warmed, saline solution were used, respectively. Ultrasound was used to measure the shortest distance between a point on the median supraumbilical abdominal wall and the promontory. The distances increased during the instillation of the fluid from 12.3 to 16.6 cm and 11.8 to 15.9 cm, respectively. In both cases, the normal uterine position was effectively restored by digital pressure transrectally. There were no complications during the maneuvers. This case series demonstrates a new strategy that allowed us to reposition an IGU with minimal force and thereby reduce procedure-related risks for mother and child compared to more invasive measures.

## Discussion

When treating an IGU, it is helpful to be guided by the biomechanical causes of the problem. These factors can have a causative as well as a therapeutic significance. The following two tables list some conditions that can have a biomechanical influence on the mobility of the incarcerated retroflexed uterus (Tables 1 and 2). Some of the mentioned factors cannot be changed and thus only play a pathogenetic or diagnostic role in IGU.

**Table 1:** List of agonistic factors, helping to prevent or reduce a retroflexed or retroverted uterus during pregnancy

Agonistic factors
Restoring forces of the stretched vagina
Restoring forces of the stretched paracervical tissue
Reduction of the uterine volume
Reduction of the bladder volume (*)
Enlargement of the intestinal volume distal to the uterine fundus (*)

Factors labeled with an asterisk (*) are usually used with therapeutic intent

**Table 2:** List antagonistic factors, with an elevated risk for a retroflexed or retroverted uterus in pregnancy

Antagonistic factors
Adhesions of the uterine to the parietal peritoneum (†)
Fluid in the maternal bladder (*)
Limited space cranially of the pelvis/tense maternal abdominal wall (†, ‡)
Anatomy of the uterus leading to a deviation from the spherical shape (e.g., myomas, fetal peculiarities, congenital anomalies) (†)
Volume of the uterus/rapid increase in size of the uterine fundus (e.g., hemorrhage/hematoma) (†)
Absence of organs or fluids to replace the volume of the uterine fundus in the Douglas space during attempts of reduction (†, ‡)

^*^: Factor usually taken into account with therapeutic intent in conservative treatment

^†^: Factors that can traditionally only be influenced by surgical measures

^‡^: Factors that can be influenced by our method

The conservative measures in reducing an IGU support the agonistic factors. The first measure for any therapeutic approach is to increase the space required for the uterus to rotate. This can be achieved by emptying the bladder and by repositioning the patient and subsequently the uterus [12]. Raising the pelvis in a dorso-anterior position (e.g., knee–elbow position) also causes an increase in the distance between the abdominal wall and the spine due to the weight acting on the abdominal wall. Due to the minimal interference with the patient, these maneuvers are often used as the first methods of choice. If unsuccessful, further techniques can be applied to reduce the volume in the pelvis. Additional pressure can be applied to the uterine fundus to induce a reduction of the IGU [11]. This can be done by finger pressure either vaginally or rectally when the patient is either in a dorsal lithotomy, knee–chest, or on all fours position [9, 13]. If this is also unsuccessful, displacement of the uterine fundus from the pelvis can be supported by filling the rectum [14]. Either a gas or a liquid can be used for this. Liquid media offers the advantage of being less likely to leave the rectum cranially (by passing uterus). Unlike gases, liquids are not compressible and therefore do not change their volume under the pressures applied. In our experience, ultrasound gel has proven effective because of its viscosity. Another therapeutic approach is to reposition the uterine fundus by applying manual pressure transabdominally [15]. In addition, strengthening the elastic restoring forces of the vagina can, at least theoretically, be another possible therapeutic approach. Here, transvaginal traction can be applied to the uterine cervix in a caudal direction [13]. It is possible to combine the mentioned conservative techniques to reduce an IGU.

**Fig. 5:**
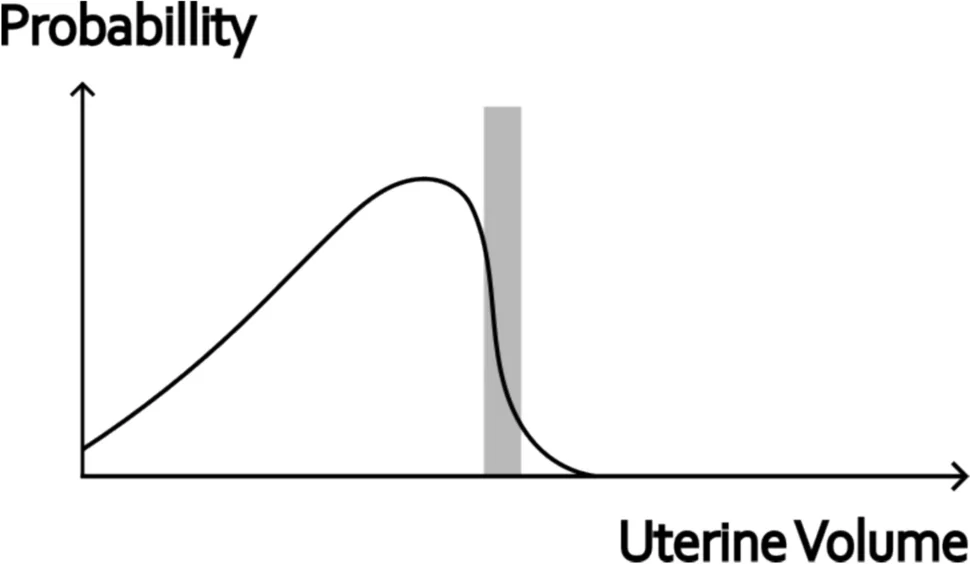
Probability of reduction as a function of uterine volume (and thus gestational age). The probability of successful uterine rotation (spontaneous or through interventions) can only be significantly increased in the gray area. A reduction of the uterine volume can then be considered as a treatment option. Schematic representation with arbitrary axis scales

As an alternative to therapeutic efforts, which strengthen the agonistic factors, weakening the antagonistic factors in their effectiveness is an option. For a few of these factors, this can only be done surgically (see † in Table 2) and, therefore, should be a reserved solution when the non- or minimally invasive techniques have failed. With the increasing volume of the uterus during pregnancy, elastic restoring forces of the uterine suspension become increasingly important. There is initially a coinciding increase in the probability of spontaneous reduction of the uterus. However, when a critical uterine volume is reached, which varies due to anatomical conditions, the probability decreases sharply. Therefore, some sources mention optimal IGU management conditions until about 20 weeks of gestation [11, 16]. If the uterine volume increases even further, a spontaneous reduction is no longer possible and the success rates of therapeutic attempts decrease depending on the uterine volume. The dependence of the probability of spontaneous or induced rotation of the uterus into the normal position as a function of the uterine volume is shown schematically in Fig. 5. With a few exceptions (e.g., polyhydramnios or rapidly progressive hematomas [17]), uterine volume is predominantly dependent on the gestational age. In a limited period during pregnancy, the possibility of uterine rotation could be relevantly improved by volume reduction of the uterus, for example, by amnioreduction [12]. At a later point in time during the pregnancy, a volume reduction that is tolerable for the pregnancy can no longer achieve a meaningful improvement in the success rate of the reduction.

**Fig. 6:**
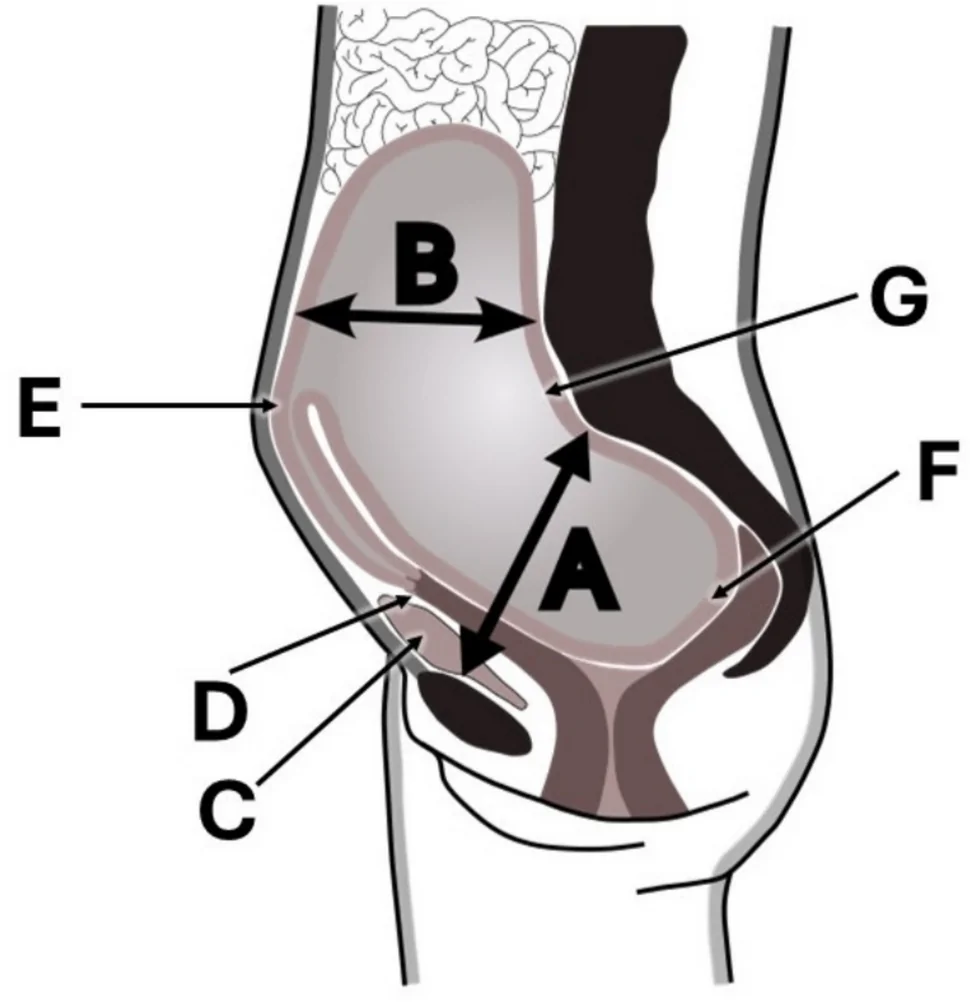
Schematic sagittal view with retroflexed uterus in pregnancy. **A** Conjugata vera/true conjugate, **B** smallest distance from the abdominal wall to the spine, **C** urinary bladder, **D** stretched vagina with external cervix, **E** internal cervix, **F** fundus of the uterus, and **G** anterior uterine wall. Distance “B” is smaller than distance “A” and thus limits the possibility of uterine rotation. The level of “B” cannot be bypassed by bowel loops of the small intestine, causing negative pressure in the pouch of Douglas during any attempt to dislocate the uterus from the true pelvis

Another risk factor is the limited space cranially of the pelvis, which prevents the rotation of the uterus. The limiting surface available for rotation of the uterus may not be the bony pelvis but the plane cranial to the pelvis (see Fig. 6). By filling the maternal abdomen with fluid, this area can be enlarged and thus facilitate the rotation of the uterus. Filling the abdomen with gas is not a viable option due to the significant interference of gas with the necessary ultrasound imaging during the procedure to monitor the fetal health, as well as the risk of inducing a maternal air embolism. Furthermore, the mechanical properties of gas lead to high compressibility, which limits its ability to produce sufficient space for uterine rotation.

The last risk factor that can be exploited therapeutically without surgery is the impossibility of a volume shift into the true pelvis to counteract a negative pressure between the uterine fundus and the peritoneum in the true pelvis. This factor has not been considered in the literature so far. In our opinion, it has increasing importance in the treatment of an IGU with increasing gestational age. Above a critical uterine volume, it is impossible for the organs located cranially to the uterus to replace the volume of the corpus uteri in the true pelvis when the uterus repositions. To do so, these organs would have to pass caudally through the area of the pelvic entrance plane. Since this area is almost completely filled by the uterus, this cannot occur with a large uterine volume as there is usually no gas and only very limited fluid in the abdominal cavity physiologically. Filling the rectum also does not provide enough volume in the true pelvis to replace the volume of the uterine corpus. Any attempt to displace the uterine corpus out of the pelvis inevitably results in negative pressure in the space between the uterine fundus and the parietal peritoneum and prevents a reduction. This phenomenon is well known from a clinically common situation: the development of the fetal head during a cesarean section, when the fetal head has already entered deep into the maternal pelvis. The aim of our intervention is to avoid the development of negative pressure by allowing a fluid to fill the gap between the uterine fundus and the parietal peritoneum. In principle, the direct application of a fluid into this gap would be possible, e.g., transvaginally. Due to the difficulty of finding this gap and the associated higher risk of infection, we decided against this approach and preferred puncturing the upper abdomen. This procedure is routine in laparoscopy and is characterized by very low known risks. Initially, fluid induction into the abdomen resulted in a significant increase in the distance between the abdominal wall and the spine. This increased the surface area available for rotation of the uterus. As an influence of one of the antagonistic factors, this mechanism can facilitate uterine rotation alone. Furthermore, the fluid is able to flow along the uterus into the true pelvis. This negated the negative pressure in the gap between the uterus and the peritoneum in the true pelvis. In addition to the typical positioning of the patient in the knee–elbow position, a small amount of pressure applied to the uterine fundus transrectally was sufficient to achieve positional normalization of the uterus in our cases.

In summary, our method increases the available surface area for uterine rotation at the most critical point and, on the other hand, avoids the negative pressure that occurs when attempting to rotate the uterus in the true pelvis. These two factors have not been considered in previously published methods.

Our technical report is based on two cases. As with any intervention, assessing a patient’s prior medical history for exclusion criteria, including multiple abdominal surgeries or suspected intraabdominal adhesions, is essential for ensuring a safe procedure. In addition, the patient must provide informed consent by being aware of the potential risks of the treatment. This procedure is similar to the abdominal puncture required during laparoscopic surgery, a method whose safety is well established. The intraabdominal application of saline solution over a short period of time is also considered generally safe. Nevertheless, possible complications of this method are similar to those of laparoscopy, including but not limited to organ injury, bleeding, infection, wound complications, anesthesia-related complications, and effects of a large volume of intraperitoneal fluid, such as electrolyte shifts, and hemodynamic or respiratory effects. Furthermore, the operator’s experience and expertise are also important considerations when applying this technique. To carry out this intervention, the treating physician should be proficient in minimally invasive surgical techniques to be able to apply intraabdominal fluid, as well as fetal monitoring to assess the well-being of the fetus during the procedure. Experience repositioning a retroflexed uterus or external cephalic version when managing a breech presentation may be helpful.

If retroflexed incarceration of a uterus during pregnancy cannot be reduced by simple conservative measures, our intervention provides a nonsurgical alternative before considering more invasive measures. Based on biomechanical principles, this approach minimizes stress for both the patient and the fetus. Applying significant force to the uterus—and the associated risk of obstetrical complications—can thus be avoided. If this method is unsuccessful, surgical intervention may be necessary.

## Data Availability

No datasets were generated or analyzed during the current study.
